# Using a simple rope-pulley system that mechanically couples the arms, legs, and treadmill reduces the metabolic cost of walking

**DOI:** 10.1186/s12984-021-00887-3

**Published:** 2021-06-07

**Authors:** Daisey Vega, Christopher J. Arellano

**Affiliations:** Department of Health and Human Performance, Center for Neuromotor and Biomechanics Research, University of Houston, 3875 Holman St., Rm 104 Garrison, Houston, TX 77204-6015 USA

**Keywords:** Locomotion biomechanics, Walking, Assistive device, Energetics, Arms, Legs, Coordination, Gait rehabilitation

## Abstract

**Background:**

Emphasizing the active use of the arms and coordinating them with the stepping motion of the legs may promote walking recovery in patients with impaired lower limb function. Yet, most approaches use seated devices to allow coupled arm and leg movements. To provide an option during treadmill walking, we designed a rope-pulley system that physically links the arms and legs. This arm-leg pulley system was grounded to the floor and made of commercially available slotted square tubing, solid strut channels, and low-friction pulleys that allowed us to use a rope to connect the subject’s wrist to the ipsilateral foot. This set-up was based on our idea that during walking the arm could generate an assistive force during arm swing retraction and, therefore, aid in leg swing.

**Methods:**

To test this idea, we compared the mechanical, muscular, and metabolic effects between normal walking and walking with the arm-leg pulley system. We measured rope and ground reaction forces, electromyographic signals of key arm and leg muscles, and rates of metabolic energy consumption while healthy, young subjects walked at 1.25 m/s on a dual-belt instrumented treadmill (n = 8).

**Results:**

With our arm-leg pulley system, we found that an assistive force could be generated, reaching peak values of 7% body weight on average. Contrary to our expectation, the force mainly coincided with the propulsive phase of walking and not leg swing. Our findings suggest that subjects actively used their arms to harness the energy from the moving treadmill belt, which helped to propel the whole body via the arm-leg rope linkage. This effectively decreased the muscular and mechanical demands placed on the legs, reducing the propulsive impulse by 43% (*p* < 0.001), which led to a 17% net reduction in the metabolic power required for walking (*p* = 0.001).

**Conclusions:**

These findings provide the biomechanical and energetic basis for how we might reimagine the use of the arms in gait rehabilitation, opening the opportunity to explore if such a method could help patients regain their walking ability.

*Trial registration:* Study registered on 09/29/2018 in ClinicalTrials.gov (ID—NCT03689647).

**Supplementary Information:**

The online version contains supplementary material available at 10.1186/s12984-021-00887-3.

## Background

The mechanical and neural benefits that stem from the natural coordination of the arms and legs during walking have inspired scientists and practitioners to emphasize this natural behavior during gait rehabilitation [1–9]. For instance, Behrman and Harkema [2] were the first to exploit the benefit of coordinating the arms' swinging motion with the stepping motion of the legs during treadmill training with body weight support [1, 2]. In a series of case studies, physical therapists would instruct patients to intentionally swing their arms or facilitate their arm motion with hand-held poles [2]. Alternatively, a recumbent stepper or cycle ergometer can allow for an individual to actively coordinate their arm and leg movements while remaining seated. As opposed to the passive arm motion facilitated by hand-held poles, these devices have the added benefit of putting the patient in control. Through this process, individuals can use their arms to modulate the amount and timing of assistance that help drive the motion of their legs, thereby becoming actively engaged in their gait re-training. Experiments studying the training effects of actively coordinating the arms and legs with these devices have shown functional improvements in walking performance in individuals with incomplete spinal cord injury [10] or chronic stroke [11, 12]. The improvements seen in recumbent stepping and cycling may have arisen from exploiting the neural coupling that underlies the coordinated motion of the arms and legs [2–4].

While recumbent stepping and cycling have shown benefits, a notable concern is that this type of activity lacks some gait-related task specificity [13]. For example, the recumbent stepping and cycling kinematics of the hip, knee, elbow, and shoulder joints are fundamentally different from walking [5, 13]. Additionally, these devices do not allow the lower limbs to undergo continuous loading and unloading, as done during treadmill training with body weight support. The act of rhythmically loading and unloading the legs is recognized as a critical sensory cue for promoting walking recovery during gait rehabilitation [14, 15]. In order to promote walking recovery, the training task should have similar sensory cues as the goal task [2, 16]. Therefore, developing a strategy where an individual can simultaneously benefit from actively coordinating the arms and legs (as done in recumbent stepping and cycling) and rhythmically loading and unloading the leg during treadmill walking could further optimize task specificity and enhance walking recovery. However, an approach that allows an individual to actively use their arms to drive the motion of their legs during treadmill walking has remained elusive.

**Fig. 1:**
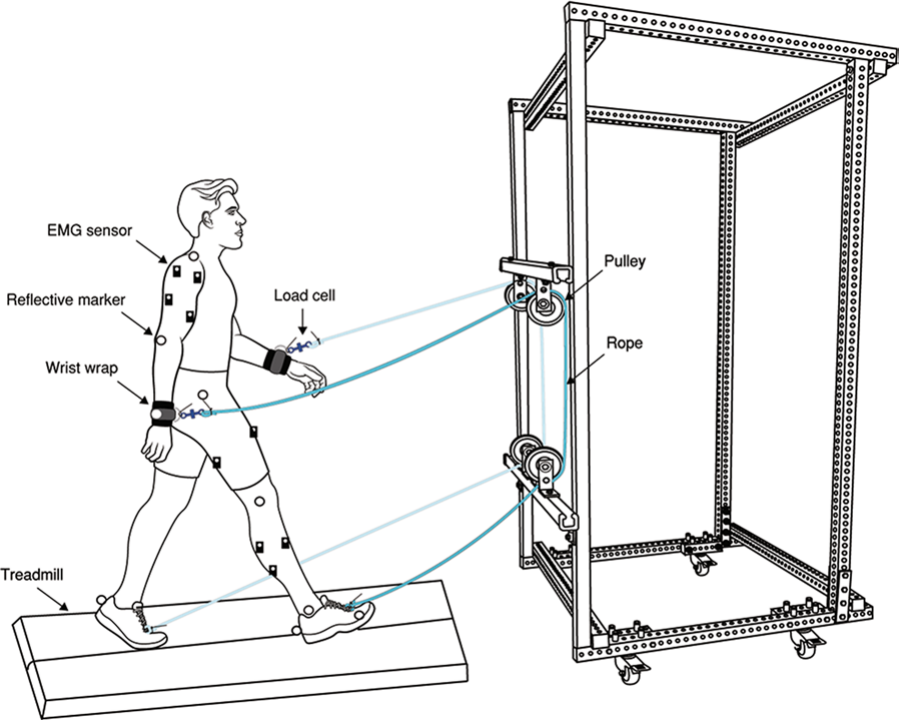
Arm-leg rope pulley system. Subjects walked on a split-belt force measuring treadmill while attached to a simple device that connects the ipsilateral arm and leg using a rope. The horizontal pulley bars are height adjustable, allowing for relative changes in rope length. Furthermore, the load cell is in series with the rope and used to measure rope tension during treadmill walking. Note that the reflective markers were attached to both sides while EMG sensors were placed only on the right side of the body due to a limited number of sensors available in our lab

To explore this idea, we developed a rope-pulley system that physically links the ipsilateral arm and leg during treadmill walking (Fig. 1). With this approach, we imagined that individuals could use their arms to assist the legs, allowing them to be more actively engaged in their gait re-training. This approach would require a greater demand from the arms, but we suspected this would lower the demand placed on the legs. Therefore, we set out to establish proof-of-concept by first understanding how our method of linking the arms and legs would influence the mechanical, muscular, and metabolic demands of walking in a cohort of healthy, neurologically-unimpaired individuals. We presumed that a user could pull on the rope as the arm swings backward and, thus, generate a force along the rope to assist with ipsilateral leg swing. We reasoned that increasing the muscular demand of the arms would incur a metabolic cost; however, if the assistive force is transmitted effectively along the rope, we expected this assistive force to decrease the muscular and metabolic demand to swing the leg, which is estimated to comprise between 10 and 33% of the net metabolic cost of walking [17–19]. Given our logic, we hypothesized that (1) walking with the arm-leg pulley system would increase the arm’s muscular demand to generate an assistive force, but a trade-off would be a decrease in the leg’s muscular demand to swing the leg, and (2) any metabolic cost incurred to actively use the arms would be counterbalanced by the reduction in the cost to swing the legs, bringing about no change in the net metabolic cost of walking. A test of our first hypothesis would provide proof-of-concept that it is possible to actively use the arms to move the legs during treadmill walking. A test of our second hypothesis would give insight into whether this approach comes at the expense of an increased metabolic cost or not. Our overall goal in this study is to provide a fundamental understanding of how physically coupling the arms and legs affects the biomechanics and energetics of walking. We believe this fundamental understanding will provide insight into its potential use as a rehabilitation strategy for individuals with impaired lower limb function, such as those recovering from a spinal cord injury or stroke.

## Methods

### Participants

Eight healthy, young subjects participated in this study (3 women and 5 men; age = 23.25 ± 3.37 years, mass = 73.88 ± 18.46 kg, height = 173.84 ± 13.95 cm; mean ± SD). Prior to the experimental session, a telephone interview was conducted to ensure the participants met the inclusion criteria of a healthy participant, i.e., physically active, non-smoker, body mass index < 30.0 kg/m^2^, and free from musculoskeletal injuries. A health screen form was then completed, reviewed, and signed by the subject the day of the experimental session. In addition, they read and signed an informed consent document. This study was reviewed and approved by the University of Houston Institutional Review Board.

### Sample size

Our sample size was based on the study of Gottschall and Kram [17], where they used an external leg swing assist apparatus to quantify the cost of leg swing during human walking (*n* = 10). Therefore, we set out to obtain a sample size of 10 subjects. However, we were limited to 8 subjects due to the malfunction of the miniature load cells placed at the wrists, which were not easily replaceable within a reasonable time frame.

### Description of the arm-leg rope pulley system

We custom built a 39″ w × 39″ l × 72″ h rope-pulley device using slotted square tubing and solid strut channels (Fig. 1). Two height-adjustable bars spanned the device horizontally (upper and lower pulley bar), each consisting of two low-friction pulleys. A diamond braided nylon rope (3/16″ in diameter; SGT Knots) passed through a pair of pulleys, i.e., one rope passed through the two left-sided pulleys while another rope passed through the two right-sided pulleys. Each rope connected to a subject’s wrist via a wrist wrap at one end. At the other end, the rope connected to the subject’s foot via shoelaces and was secured using heavy duty cable ties. A subminiature load cell (Model: LCM201-500; Omega Engineering, Stamford, CT, USA) was placed in series with each rope and wrist wrap on the right and left side to measure the pulling forces generated by each arm during walking. Our rationale for implementing this set-up was based on our idea that the arm would generate an assistive force during arm retraction. Given that the maximum retraction of the right arm corresponds with right heel strike [20], our intent was to allow the arm to provide an assistive force via the rope to swing the ipsilateral leg forward.

#### Device protocol

During pilot testing, we developed a standard protocol to attach the device to each subject (see Additional file 1). The primary goal of our standard protocol was to minimize any slack along the rope so that any pulling force generated by the arm would pull the leg as naturally as possible. First, the horizontal bars on the device were adjusted to each subject’s height by raising the height of the upper bar to shoulder level and the height of the lower bar to knee level. Subjects were then instructed to stand in the middle of the treadmill, face the device and extend one arm until a 20-degree angle was formed with respect to the vertical axis (measured with a hand-held goniometer). At this position, we attached one end of the rope to the subject’s wrist, passed the rope along the top and bottom pulley, and then attached the other end of the rope to the shoelace of the ipsilateral foot. This procedure was repeated on the other side. Subjects were then given a short practice session (~ 2–3 min) and instructed to actively use their arms to pull on the rope to assist their legs while walking. During this practice session, the treadmill speed started at 0.25 m/s. Once the subject felt comfortable and capable of continuing, the speed was manually incremented by 0.25 m/s until they reached the speed of 1.25 m/s. All subjects successfully reached this speed and were allowed rest ad libitum before continuing with the remainder of the experiment.

### Experimental measures

Prior to the experimental trials, subjects performed a 7-min standing trial while we measured rates of oxygen consumption and carbon dioxide production to estimate standing metabolic power. We then compared the mechanical, muscular, and metabolic demands of healthy individuals (n = 8) walking on a treadmill (1.25 m/s) during two randomized conditions of normal (control) and assisted walking. In the normal condition, subjects walked without the rope-pulley device. In the assisted condition (Fig. 1), subjects were connected to the device and were instructed to use their arms to pull on the rope to drive their legs. Subjects walked on a dual-belt treadmill (Bertec Corporation, Columbus, OH, USA) at 1.25 m/s for the randomized trials of normal and assisted walking, with each trial lasting 7 min in duration. Subjects were allowed a full recovery ad libitum of at least 5 min between trials to reduce any effects of fatigue. During both trials, we simultaneously measured ground reactions forces (GRFs), positions of reflective markers, surface electromyography (EMG), rates of oxygen consumption ($\dot{V}{\text{O}}_2$) and carbon dioxide production ($\dot{V}{\text{CO}}_2$), and respiratory exchange ratio (RER) during the last three minutes of each trial. During the assisted condition, we measured the pulling forces generated by the arm by means of subminiature load cells.

#### Kinematics and kinetics

Prior to each data collection, we obtained anthropometric measurements of body segment lengths and placed reflective markers on the lower and upper extremities using a simple body marker set, similar to Arellano and Kram [21]. In addition, reflective markers were placed on the arm-leg rope pulley system. Our twelve-camera motion capture system was calibrated using a 5-marker wand and a standard L-Frame (Vicon, Oxford, UK). Prior to each trial, we recorded a 5-s standing calibration to use as a baseline for GRFs and an anatomical reference frame for each segment. In addition, we measured the baseline of the load cells at rest. Prior to the study, each load cell was calibrated with known weights ranging from 0 to 444.82 Newtons. The slope of the calibration regression line was used to convert the voltage output into force expressed in Newtons. Each load cell was placed on the left- and right-sided rope connection. However, due to hardware issues, we were only able to record load cell data on the right side for seven subjects. The GRF and load cell data were sampled at 1000 Hz while the 3D positions of the reflective markers were sampled at 100 Hz.

#### Muscle activity

Muscle activity of the right upper and lower limbs were measured through surface EMG sensors (Delsys Inc, Natick, MA, USA). Prior to the experiment, we prepped the subject’s body for EMG sensor placement by shaving the skin to remove any hair, followed by scrubbing and cleaning the skin with an alcohol wipe. Sensors were placed only on the right upper and lower limb muscles using the recommendations from SENIAM (seniam.org/sensor_location.htm). After sensor attachment, we secured them with elastic tape to minimize movement artifact. The sampling rate was set to 2000 Hz, and the activity of the following muscles were measured: posterior deltoid (PD), anterior deltoid (AD), long head triceps brachii (TRI), biceps brachii (BIC), bicep femoris (BF), rectus femoris (RF), soleus (SOL), medial gastrocnemius (MG), and tibialis anterior (TA). Given that we were limited to nine EMG sensors in our lab, we identified four upper limb muscles and five lower limb muscles to evaluate in our study. Based on previous studies that partitioned the role of specific muscles during walking, we identified muscles based on their role in the stance and swing phase of walking. Following the convention of Gottschall and Kram [17], we analyzed potential changes in the EMG activity of the BF, RF, and TA as muscles that contribute to the swing phase. On the other hand, we analyzed the MG and SOL as muscles that contribute to the propulsive period of the stance phase and the BF, RF, SOL, MG, and TA as muscles that contribute to the braking period of the stance phase [17, 22, 23]. And finally, we analyzed potential changes in the EMG activity of all the upper limb muscles (PD, AD, TRI, and BIC) during all three phases. While the arms do not play a direct role in generating forces along the ground during normal walking, the PD appears to play a primary role in arm swing retraction [20], while the AD, TRI, and BIC seem to play a trivial role in facilitating the swinging motion of the arms [24]. Nonetheless, we expected that if an individual used their arms to generate an assistive force while using our device, the muscular effort would come from the shoulder and upper arm muscles.

#### Metabolic energy

We collected metabolic data through an indirect calorimetry system that measures $\dot{V}{\text{O}}_2$, $\dot{V}{\text{CO}}_2$, and RER (ParvoMedics TrueOne 2400, Sandy, UT, USA). Throughout each trial, including quiet standing, we monitored that RER values were between 0.7 and 1.0 to confirm that the metabolic energy was primarily being supplied through aerobic metabolism. Prior to each data collection, a 3L flowmeter and gas calibration with known gas concentrations were performed.

### Data analysis

Using a custom code in MATLAB (MathWorks Inc., Natick, MA, USA), we compiled the GRFs, load cell, positions of reflective markers, and EMG data during the last 3 min of each trial. We performed data analysis over 10 consecutive walking gait cycles that were free from movement artifact and only analyzed the GRF of the right leg. The GRF data was filtered using a zero-phase, 4th order Butterworth filter with a frequency cut off of 20 Hz. From the vertical GRF, our algorithm identified instances of heel strike and toe off. A gait cycle was defined as right heel strike to the next right heel strike. For each gait cycle, we computed impulse by computing the integral of force (N) with respect to time (s) using the trapezoidal method for the vertical, horizontal, and load cell data. Braking and propulsive impulses were calculated by integrating all of the negative and positive values from the horizontal GRF data, respectively. The impulse values were then averaged among the 10 gait cycles and then normalized by body weight (BW) for each subject. The load cell data was processed in two steps: (1) the baseline offset was removed, and (2) voltage was converted to newtons by using the slope of the calibration regression line. The load cell data were then averaged among the 10 gait cycles and then normalized to body weight for each subject.

The 3-D positions of the reflective markers were first splined to adjust for any missing gaps during the digitization process and then filtered using a zero-phase, 9th order Butterworth filter with a frequency cut off of 6 Hz. The relative joint angles were computed in the sagittal plane using Winter’s method [26]. The following joint angles were computed (with its respective body segments): shoulder (upper arm and trunk), elbow (upper arm and forearm), hip (trunk and thigh), knee (thigh and shank), and ankle (shank and foot). For each subject, the relative joint angles were expressed with respect to the 5-s standing trial, which acted as their own anatomical reference frame.

The EMG signals were downsampled to 1000 Hz, its baseline offset removed, and filtered with a bandpass of 20 to 450 Hz. Following Yang and Winter [25], we computed the linear envelope for each signal in a two-step process: (1) a full-wave rectification and (2) a zero-phase, low-pass 2nd order Butterworth filter with a frequency cutoff of 6 Hz. For each gait cycle, we computed the average EMG (aEMG) for each muscle during the braking, propulsive, and swing phases of walking (e.g., aEMG of PD during braking, aEMG of PD during propulsive, aEMG of PD during swing, aEMG of AD during braking, etc.). Using Winter’s method [26], the aEMG was computed by the integration of the signal with respect to time and then dividing by the total time duration. The aEMG for each muscle and phase of walking was then averaged over 10 gait cycles for each subject and then normalized to the aEMG value of the control trial (the aEMG value of its respective muscle and phase during normal walking). In this case, the normalized aEMG value of each muscle during each phase of normal walking is equal to 1, whereas the normalized aEMG value of each muscle during each phase of assisted walking was less than, equal to, or greater than 1.

To create each subject’s ensemble curves (see Additional file 2: Figure S1), each dependent variable was splined over each gait cycle to be expressed as 0% to 100% of a gait cycle, averaged across all 10 gait cycles (ensemble average), and normalized as described above. We quantified the mean ensemble curves by averaging the subject ensemble curves.

For the last 3 min of each trial, we averaged the RER values and calculated metabolic power from the weighted average $\dot{V}{\text{O}}_2$ and $\dot{V}{\text{CO}}_2$ using the Brockway equation [27]. Net metabolic power was obtained by subtracting the standing metabolic rate from the walking metabolic rate of each trial.

### Statistical analysis

The assumption of normality for each variable was tested using the Kolmogorov–Smirnov test. Standard paired *t*-tests were performed on variables that met the assumption of normality; otherwise, the non-parametric Related Samples Wilcoxon Signed Rank test was used to test for significant differences between normal and assisted walking conditions. For all dependent variables, we compared assisted walking against normal walking using a two-sided test, except for the aEMG activity of the muscles during the swing phase. We presumed that when using the device, subject’s would use the arms to generate an assistive force to help swing the leg, which would reflect an increase in arm muscle activity (AD, PD, TRI, and BIC) and a decrease in leg muscle activity (BF, RF, and TA). Therefore, we tested for significant differences in muscle activity during the swing phase using a one-sided paired *t*-test or a one-sided Related Samples Wilcoxon Signed Rank test. All statistical tests were run with an alpha level of 0.05 (IBM SPSS Inc., Chicago, IL, USA). The statistical table for the biomechanical and metabolic data can be found in Table 1. In addition, all statistical tables for the aEMG data can be found in Supplementary Tables 1–3 (see Additional file 3: Table S1). In the tables, we provide exact *p* values. While in the main text, we include when *p* is less or greater than 0.05 unless noted otherwise.

**Table 1:** Biomechanical and metabolic data for subjects

Variable	Normal mean ± SD	Assisted mean ± SD	% change	*n*	*p*
Net metabolic power (W/kg)	3.04 ± 0.58	2.53 ± 0.38	− 16.78*	*n* = 8	0.001
Gross metabolic power (W/kg)	4.38 ± 0.59	3.87 ± 0.36	− 11.64	*n* = 8	n/a
Gross *V̇*O_2_ (L/min)	0.921 ± 0.133	0.832 ± 0.156	− 9.67	*n* = 8	n/a
Normalized (L/min/kg)	0.013 ± 0.002	0.012 ± 0.001	− 7.69	*n* = 8	n/a
Respiratory exchange ratio	0.85 ± 0.04	0.80 ± 0.06	− 5.88	*n* = 8	0.077
Stride frequency (Hz)	0.91 ± 0.08	0.90 ± 0.08	− 1.10	*n* = 8	0.121
Vertical impulse (N·s)	404.47 ± 120.17	404.80 ± 116.13	0.08	*n* = 8	0.962
Normalized (N·s/BW)	0.5523 ± 0.016	0.5538 ± 0.017	0.27	*n* = 8	0.842
Braking impulse (N·s)	− 24.55 ± 7.09	− 36.28 ± 12.14	47.78*	*n* = 8	0.001
Normalized (N·s/BW)	− 0.0337 ± 0.001	-0.0492 ± 0.002	45.99*	*n* = 8	0.000
Propulsive impulse (N·s)	24.63 ± 7.36	13.53 ± 4.33	− 45.06*	*n* = 8	0.003
Normalized (N·s/BW)	0.0337 ± 0.001	0.0192 ± 0.002	− 43.03*	*n* = 8	0.000
Assistive impulse (N·s)	n/a	16.39 ± 10.21	n/a	*n* = 7	n/a
Normalized (N·s/BW)	n/a	0.0208 ± 0.001	n/a	*n* = 7	n/a
Peak assistive force (N)	n/a	52.05 ± 21.99	n/a	*n* = 7	n/a
Normalized (% BW)	n/a	6.93 ± 1.81	n/a	*n* = 7	n/a

All comparisons were made between normal and assisted walking conditions with significance at *P* < 0.05. *Signifies significant differences between walking conditions. All comparisons were tested at an alpha level of 0.05 using a two-sided, paired samples t-test. n/a = not applicable

## Results

### Kinetics and kinematics

**Fig. 2:**
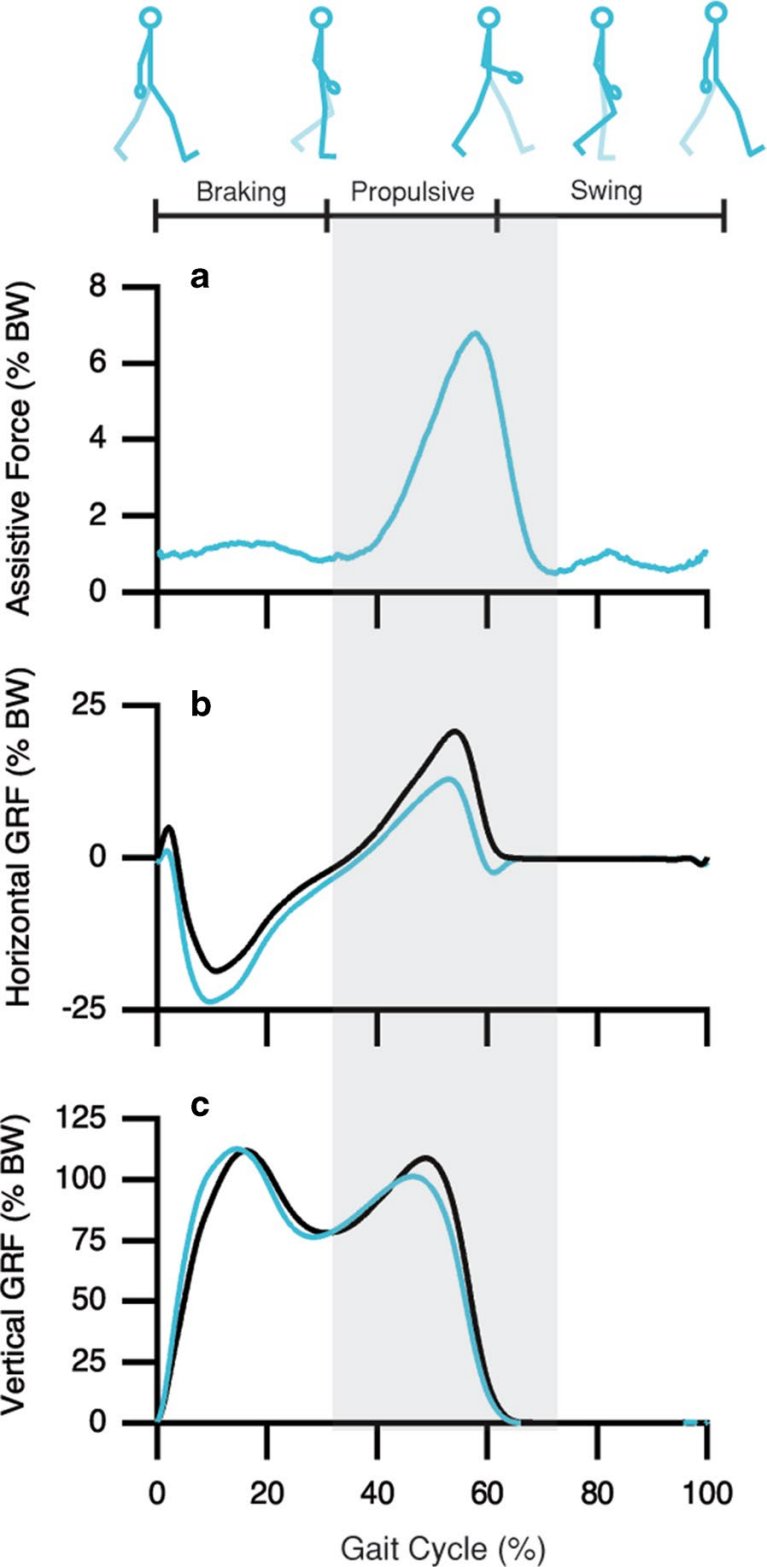
Mean ensemble forces demonstrating the mechanical demands during both normal (black line) and assisted (blue line) walking conditions. **a** During ~ 30–70% of the walking gait cycle, an assistive force was generated by the same-sided arm and leg rope connection (shaded gray area; *n* = 7). This assistive force is best understood as a forward force applied to the whole body. **b** In turn, this caused a decrease in propulsive and an increase in braking forces generated by the leg during assisted walking as compared to normal walking (*n* = 8). **c** The vertical GRF, which is required to support and accelerate the body, remained the same during both conditions (*n* = 8). Note that an assistive force was generated by the right arm and leg rope connection and another assistive force by the left arm and leg rope connection, but we only illustrate the assistive force generated by the right side

The assistive force was generated between ~ 30 and ~ 70% of the gait cycle and peaked at 60% (Fig. 2a). Over an entire gait cycle, the assistive force, i.e., the tension produced by a single physical connection of the same-sided arm and leg, applied a total impulse of 0.0208 N·s/BW with an average peak force of 7% of body weight. For all subjects, the increase in assistive force coincided with an increase in shoulder protraction (see Additional file 2: Figure S1). However, the change in elbow joint angle varied; some subjects underwent elbow flexion, others underwent extension, and some kept the angle relatively fixed (see Additional file 2: Figure S1). Despite the variation in elbow joint behavior during force rise, the assistive force elicited a 43% decrease in the propulsive impulse and a 46% increase in the braking impulse generated by the leg (measured by the horizontal GRF; two-tailed *p’s* < 0.001; Fig. 2b). Furthermore, there was no change in either vertical impulse (two-tailed *p* = 0.842; Fig. 2c) or stride frequency (two-tailed *p* = 0.121).

### Muscle activity during assisted walking

#### Braking phase (Fig. 3a)

**Fig. 3:**
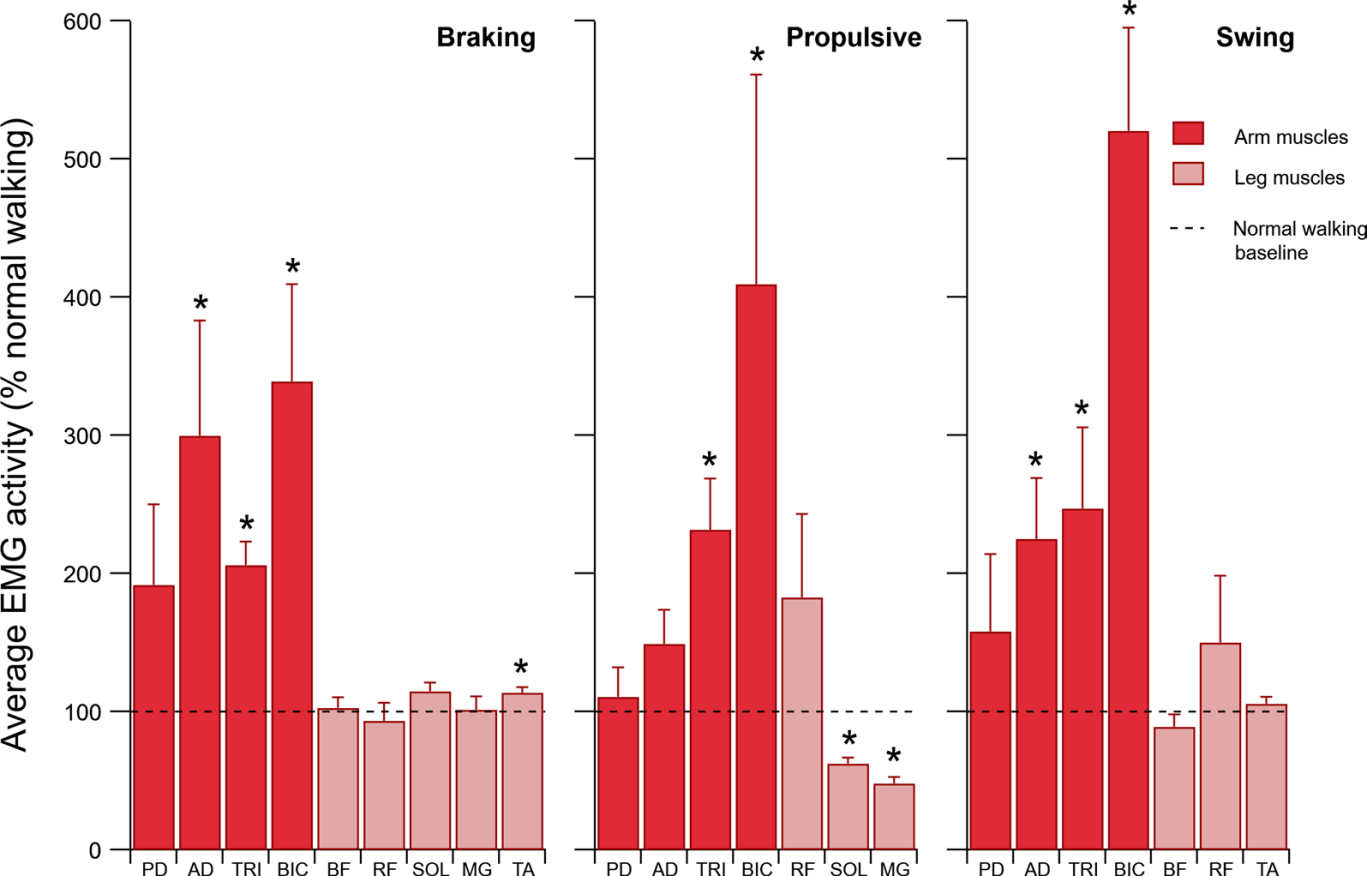
Average electromyographic (aEMG) activity during assisted walking. We assessed a total of four upper and five lower limb muscles (mean ± SE; *n* = 8; see list of abbreviations). Each value is expressed relative to normal walking, representing a baseline of 100% (dashed black line). This data reveals a muscular shift characterized by greater arm and lesser leg muscle activity. Most notably, the arm’s triceps and biceps were the primary muscles to help transmit the assistive force onto the whole body during the propulsive phase, which reduced the leg’s medial gastrocnemius and soleus demand. * indicates a significant difference from baseline, *p* < 0.05

The aEMG activity of the TRI, BIC, and AD increased by 106%, 239%, and 200%, respectively (two-tailed *p’s* < 0.05). At the same time, the aEMG activity of the TA anterior increased by 14% (two-tailed *p* = 0.012).

#### Propulsive phase (Fig. 3b)

The aEMG activity of the TRI and BIC increased by 132% and 309%, respectively (two-tailed *p*’s < 0.05). For the leg muscles, the aEMG of MG and SOL decreased by 52% and 38%, respectively (two-tailed *p’s* < 0.001).

#### Swing phase (Fig. 3c)

The aEMG activity of the TRIC, BIC, and AD increased by 147%, 420%, and 125%, respectively (one-tailed *p*’s < 0.05). In the leg muscles studied here, we found no decrease in the aEMG activity of the RF, BF, or TA (one-tailed *p*’s > 0.05).

### Metabolic energy

**Fig. 4:**
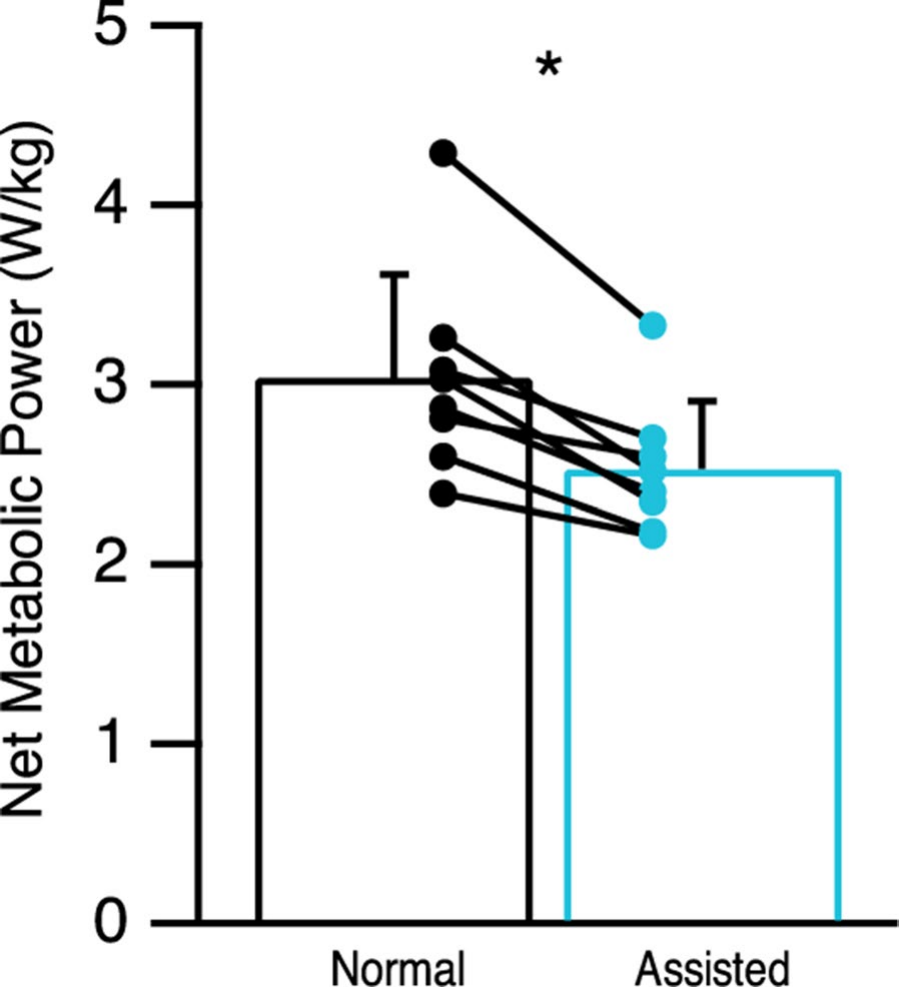
Net metabolic power demand for walking. Using the arm-leg pulley system reduced the net metabolic power required to walk by 17% (mean ± SD,* n* = 8). Each line segment represents a subject, highlighting the observation that all subjects showed a reduction in net metabolic power (* indicates *p* < 0.05)

The net metabolic power required for normal walking (3.04 ± 0.57 W/kg) was greater than the net metabolic power required for assisted walking (2.53 ± 0.37 W/kg; *t*(7) = 5.526, two-tailed *p* = 0.001; Fig. 4). Furthermore, there were no significant differences in the RER values between normal (0.85 ± 0.04) and assisted walking (0.80 ± 0.06; *t*(7) = 2.075, two-tailed *p* = 0.077).

## Discussion

We found the arms to play a critical role in eliciting changes in the leg’s mechanical and muscular demand during the assisted walking trial. We discovered that the arm helped modulate the magnitude of the assistance force that was applied onto the whole body and not directly onto the legs. Nonetheless, the muscular demand of the arms increased while the muscular demand of the legs decreased, but this trade-off occurred during the propulsive phase and not the swing phase of walking as we originally hypothesized. As we discuss in more detail below, it appears that the arms were able to harness the energy from the motor-driven treadmill belt, which we did not foresee when conceptualizing the mechanical effects of physically linking the arms and legs with a simple rope-pulley system. Overall, we reject our first hypothesis because subjects did not actively use their arms to assist with leg swing but instead actively used their arms to assist with forward propulsion. Lastly, we found that the physical connection allowed the arm, leg, and treadmill to interact in a manner that led to a 17% reduction in the net metabolic power required to walk, leading us to also reject our second hypothesis. From these experimental findings, we reason the reduction in the net metabolic power required to walk can be primarily explained by the reduction in the need to generate propulsive forces by the legs.

### Assisted walking phases (see Additional file 4)

#### Propulsive phase (Fig. 5a)

**Fig. 5:**
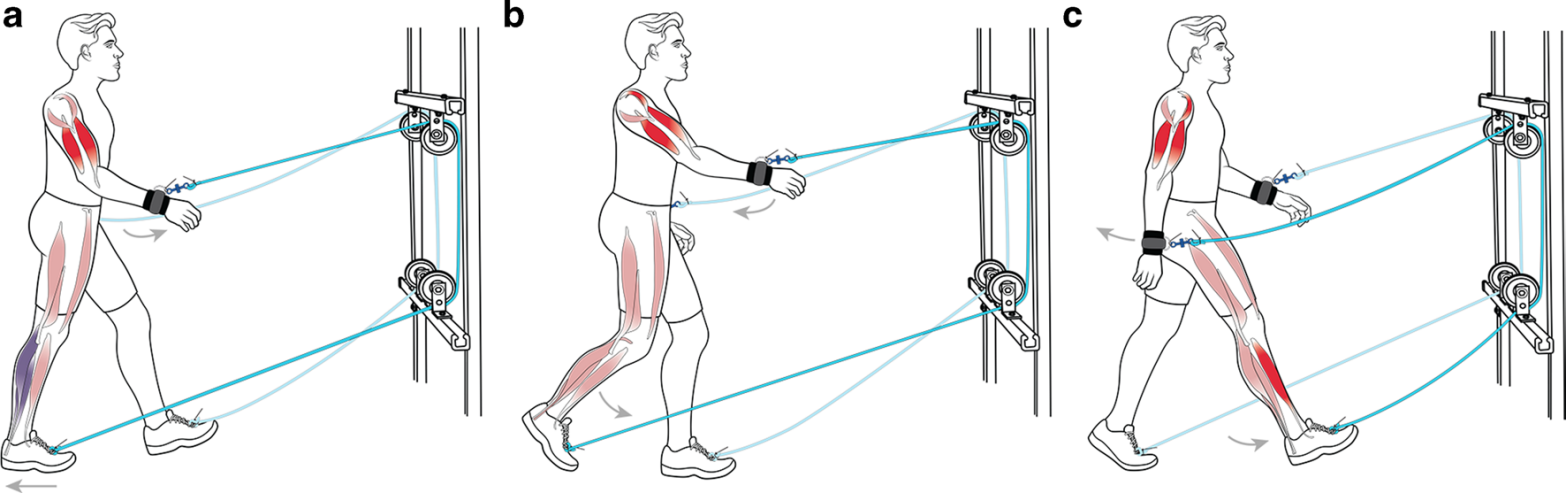
Assisted walking.** a** During the propulsive phase, the right foot is planted onto the treadmill belt, which is moved backward. In turn, this movement pulled the right arm forward. The increased muscle activity of the biceps and triceps (bright red) helped stiffen the arm, which was necessary to transmit the force onto the whole body, eliciting a net forward force. The net forward force reduced the need for propulsion from the right leg and, therefore, reduced the muscular demand of the medial gastrocnemius and soleus (purple).** b** During the swing phase, the muscle activity of the arm’s biceps, triceps, and anterior deltoid (bright red) increased, but this activity did not coincide with a decrease in leg muscle activity.** c** During the braking phase, the right arm swung backward without causing any rope tension. At the same time, the left arm was helping to transmit the assistive force onto the whole body during the propulsive phase of the left leg. This forward force generated by the left arm and leg connection decreased the need for propulsion in the left leg but increased the demand for braking in the right leg. As such, an increase in the right tibialis anterior muscle activity occurred (bright red)

During the propulsive phase of normal walking, which occurs between 30 and 60% of the gait cycle, the leg generates a force to push off the ground and propel the body forward. As shown in Fig. 2a, an assistive force occurs throughout the entire propulsive phase. The rise in assistive force coincided with the leg moving backward with the treadmill belt as the foot remained planted. At the same time, the ipsilateral arm was pulled in the forward direction. A likely explanation is that subjects used the rope-pulley system to take advantage of the mechanical work performed by the treadmill motor and moving belt (similar to Sánchez et al. [28]). Therefore, as the treadmill belt pulled the foot-to-rope connection in the backward direction, it also pulled the ipsilateral wrist-to-rope connection in the forward direction. This would indicate that the mechanical energy from the moving treadmill belt was shuttled onto the rope that linked the planted foot and ipsilateral arm. Assuming that friction forces along the pulleys are negligible, the force pulling at the foot-to-rope connection would be similar to the force pulling at the wrist-to-rope connection. In general, the assistive force is best understood as a forward force applied at both the wrist-to-rope and foot-to-rope connection and thus, pulled the whole body in the forward direction. This effect is similar to applying an external force that pushes the whole body at the waist [22, 29].

While the pulling forces acted at both the foot and wrist, the lower limb kinematics of the hip and knee remained relatively the same, but the upper limb kinematics of the shoulder and elbow varied when compared to normal walking (see Additional file 2: Figure S1). Although a standard protocol for attaching the rope to the wrist and foot was the same for all subjects, our method of physically linking the arms and legs resulted in a more flexed or extended arm throughout the assisted walking trial. The rapid rise in the assistive force measured by the load cell was coupled with an increase in shoulder protraction, whereby the entire arm swung forward. Additionally, subjects underwent different elbow strategies during force rise. Four subjects underwent elbow flexion, two subjects underwent elbow extension, and one subject maintained a fixed elbow joint angle. Despite the elbow strategy used, subjects still found a way to use their arms to help propel the body.

Furthermore, we observed a simultaneous increase in the aEMG activity of the upper arm BIC and TRI muscles (Fig. 3). We interpret the coactivation of the BIC and TRI as a means to stiffen the elbow joint. The kinematic and muscular response of the arm suggests that the arm played an active role in facilitating and modulating the magnitude of the assistive force measured by the load cell at the wrist-to-rope connection. In the absence of this modulated response, the magnitude of the assistive force generated at the wrist may have emerged more abruptly, causing a jerky movement of the whole body and potentially compromising the control of balance from step to step. While our interpretation suggests that the arms were not the primary source of energy like we anticipated, the active use of the arms were still key to modulating the assistive force and providing a propelling effect on the whole body. The propelling effect reduced the need for the trailing leg to push off the ground, which effectively decreased propulsive impulse by 43% (Table 1) and was coupled with a 52% and 38% decrease in muscular demand of the MG and SOL (Fig. 3), which are two key muscles that contribute to the leg’s ability to generate propulsive impulses during walking [22, 23].

#### Swing phase (Fig. 5b)

Following the propulsive phase of normal walking, the leg leaves the ground and swings forward, corresponding to the swing phase (~ 60–100% of the gait cycle). As shown in Fig. 2a, the assistive force rapidly decreased between 60 and 70% of the gait cycle, corresponding to the period of leg swing initiation. During this time, the arm swung back (see Additional file 2: Figure S1) and was coupled with a relative increase in the aEMG activity of the TRI, BIC, and AD (Fig. 3). The increase in the TRI and BIC activity likely reflects their role in their continued coactivation at the elbow joint to modulate the assistive force as it rapidly declined during leg swing initiation. Prior to the swing phase of walking, the arm was pulled forward by the motor-driven treadmill belt, which caused the arm to undergo greater protraction. Therefore, the AD may have helped to control and stabilize the shoulder joint as it underwent a greater retraction range during the swing phase of walking. With respect to the leg muscles studied here (Fig. 3), we found no decrease in the aEMG activity of the BF, RF, or TA, which are a combination of muscles that facilitate the swinging motion of the leg [17]. We are aware that we lack EMG measurements from the iliopsoas, a hip flexor muscle that plays a key role in leg swing initiation [17]. It is possible that the assistive force may have helped to reduce the muscular activity of the iliopsoas, but this is unlikely given that both the iliopsoas and rectus femoris act synergistically to initiate leg swing. Therefore, if the arm-leg pulley system helped with leg swing initiation, then we should have observed a reduction in the aEMG of the rectus femoris, but we did not. These findings lead us to conclude that the arm-leg pulley system had little effect on swinging the leg during walking.

#### Braking phase (Fig. 5c)

Following the swing phase of normal walking, the leg contacts the ground and generates a braking force that slows down the forward motion of the body. To compensate, the trailing leg generates a propulsive force that is equal in magnitude, which helps to achieve a constant walking speed. When using the arm-leg pulley system, the braking impulse increased by 46% compared to normal walking (Table 1). Although the assistive force during this time is ~ 1% BW (Fig. 2a), recall that this assistive force represents the tension of the rope linking the right arm and leg. We cannot ignore that another assistive force is being generated by the connection of the left arm, leg, and treadmill belt. In this case, the energy from the left treadmill belt is being used to propel the body forward at both the left wrist and foot rope connection. The forward force is then distributed among both legs during double support, i.e., when both feet are in contact with the ground (~ 50–62% of the gait cycle). The assistive force will help reduce the need for propulsion by the left leg but increase the braking force generated by the right leg to maintain a constant velocity. As such, the greater braking force is a consequence of the forward force acting at the whole body and had unintentional consequences at the muscular level by inducing a 14% increase in the aEMG activity of the TA (Fig. 3). During normal walking, the TA muscle–tendon unit plays an important role in absorbing energy during the early period of the braking phase [30]. The TA functions to control the plantarflexion torque induced at the ankle joint, caused by the braking force generated by the leg as it makes contact with the ground [28]. Therefore, a greater braking force would signify greater energy-absorbing demands on the ankle, and the increased aEMG activity of the TA suggests an increase in its contractile force to meet these energy-absorbing demands.

In addition, an increase in the aEMG activity of the arm’s AD, TRI, and BIC occurred during the braking phase (Fig. 3). This seems counterintuitive given the relatively small tension on the rope (~ 1% BW). Although the mechanism is not clear, we speculate that the changes in the aEMG activity may reflect the increased range of motion of the shoulder and elbow joints, which altered the normal-like transition from arm swing retraction to protraction (see, Additional file 2: Figure S1). Increasing the aEMG activity of the AD would facilitate a rapid transition from maximum retraction to protraction in order to maintain coordination with the legs, while the BIC and TRI would act as elbow joint stabilizers.

### Metabolic energy

During normal walking, arm swing appears to incur a small metabolic cost since the backward and forward motion arises from a combination of active muscle actuation [20, 24] and passive dynamics [31–33]. Yet, the muscle activity of the arm muscles seems relatively small. At a walking speed of ~ 1.1 m/s, the average EMG activity observed in the PD remains less than 5% of its maximum voluntary contraction (MVC) [24]. Additionally, the average EMG activity remains close to zero for the AD (< 2% MVC), TRI (< 1% MVC), and BIC (< 0.5% MVC), suggesting that these muscles play an insignificant role to swing the arms. In contrast, when using the arm-leg pulley system, the active use of the arms required a 3 to 6 fold increase in the aEMG activity of the upper-limb muscles (TRI, BIC, and AD; Fig. 3) as compared to normal walking, likely incurring a metabolic cost. Yet, actively using the arms to help propel the whole body resulted in a 17% reduction in net metabolic power (Fig. 4). It should be noted that this reduction in net metabolic power was independent of the need to support and accelerate the body in the vertical direction as subjects did not change the vertical impulse that they applied to the ground. In particular, our findings suggest that any cost incurred from actively using the arms was outweighed by the decrease in cost that emerged from lowering the propulsive demands placed on the legs, yielding a net reduction in the metabolic cost of walking.

While our arm-leg rope pulley system clearly lowered the propulsive demands placed on the legs, one might find it surprising that this effect yielded a net reduction in the metabolic power required to walk despite the fact that subjects generated greater braking forces. However, it is well established that generating braking forces are metabolically cheaper than generating propulsive forces [22, 34], which is consistent with our findings. In our study, subjects used the arm-leg pulley system to generate an average peak assistive force of 7% BW, coinciding with a 43% decrease in propulsive impulse and a 46% increase in braking impulse, eliciting a 17% reduction in net metabolic power. These observations suggest that even if actively using the arms and generating greater braking forces incurred a cost, this was outweighed by the reduction in cost from generating lower propulsive forces. Therefore, the ~ 17% reduction in net metabolic power that was observed here can be primarily explained by the reduction in the leg’s mechanical demand to generate propulsive forces.

### Limitations and future directions

With the arm-leg pulley system, the arms did not directly assist with leg swing as we expected. It may still be possible to allow for the arms to assist with leg swing; however, this would require hardware modification of the current arm-leg pulley system. A clutch/gearing mechanism can be incorporated into the arm-leg pulley system to allow direct energy transfer from the arms to the legs. Alternatively, a body-worn device could test the effects of using the arms to assist leg swing without the effects of the motor-driven treadmill belt. With a body-worn device, the forces would be internal to the body and would also allow for direct energy transfer from the arms to the legs. We plan to make hardware modifications that would allow future studies to investigate if the active use of the arms can help drive leg swing during walking.

Although our device did not allow for the arms to assist with leg swing, we found the arms could still play an active role in forward propulsion. A limitation of this study is that we did not foresee individuals using their arms to take advantage of the energy from the treadmill motor/moving belt. Nonetheless, allowing an individual to use the arm-leg assistive device while walking on a treadmill could still be useful for individuals undergoing gait rehabilitation. Future experiments will help us understand whether individuals with lower limb impairments could actively use their arms in this way. As observed in recumbent stepping experiments in patients recovering from an SCI [10, 35] and cycling experiments in patients recovering from a stroke [11, 12], it may be possible for those who have retained function in their arms to learn how to actively coordinate the arm and legs during walking. The intentional use of the arms may encourage patients to engage actively (self-driven) instead of passively (externally driven), which can happen when physical therapists or even powered assistive devices execute the stepping motion. Additionally, a major benefit of our approach is that reducing the propulsive demands placed on the legs elicited a significant reduction in the metabolic cost required to walk. We recognize that compared to other interventions where subjects learned how to walk with a lower limb assistive device [36, 37], the time allowed for our participants was relatively short. It is possible that the metabolic cost savings would have been substantially larger with more training, which would allow for an adaption period. Even so, we still observed a reduction in net metabolic power. This seems beneficial given that other assistive modalities (e.g., using arm-held crutches or ankle–foot orthoses) increase the metabolic cost of walking [38]. Furthermore, the metabolic savings observed here may help to extend the duration of a gait re-training session, which would allow for increased stepping repetition that is critical for regaining walking function [2, 16].

Although our arm-leg pulley system shows promise for assisted forward propulsion and reduced metabolic cost, future experiments are necessary to understand if these findings would translate to those undergoing gait rehabilitation. We must keep in mind that this study was conducted in healthy individuals walking at a speed of 1.25 m/s, and it should be noted that this speed is much faster than the typical speeds adopted by some clinical populations. Another issue with the current arm-leg pulley system is that the effective transmission of force between the arms and legs may be altered if the person drifts backward on the treadmill. Although this was not a major issue with our subjects, it is unknown how well clinical populations would adapt to this setup. Additionally, it remains to be understood if linking the arms and legs in this way would disrupt balance. These concerns may be alleviated by integrating our arm-leg pulley device with bodyweight support, which is usually built with a fixed suspension system surrounding a treadmill. Providing body weight support with a fixed suspension system would help the patient maintain their position on the treadmill while also minimizing the need to actively maintain balance. Lastly, the greater braking forces observed here may raise the concern of lower-limb muscle injury, but this may be avoided with progressive training [39, 40]. Considering all of these factors, our future work aims to integrate body weight support with our arm-leg pulley system, which will help us understand its mechanical and metabolic effects on walking in both healthy and clinical populations.

Given that we have provided proof-of-concept of actively using the arms during treadmill walking, it is now possible to explore if integrating our approach with body weight support could further optimize task specificity and walking recovery. Bodyweight support during treadmill walking already allows for the sensory cues of rhythmic stepping, upright posture, and more normal-like joint kinematics as compared to recumbent stepping/cycling. Combining these with the arm-leg pulley system may add another sensory cue. There is compelling evidence of a neural coupled pathway between the arms and legs [3, 5, 41], and it appears that this neural coupling can only be exploited when the swinging motion of the arms are coordinated with the stepping motion of the legs [3]. Experiments in recumbent stepping suggest that the active use of the arms can help modulate this pathway by enhancing the muscle activity of the legs in both healthy and SCI patients [8, 35, 41]. Furthermore, increasing the effort of the arms through greater handle resistance leads to an increase in the neuromuscular activity amplitudes of the passively moving legs, suggesting that the neural pathway can be further modulated by arm effort [35, 41]. In those with a spinal cord injury, functional improvements in walking performance were found in these individuals undergoing recumbent stepping training [10]. It seems that exploiting this neural coupled pathway may help the spinal cord re-learn how to walk again. It is clear that in this study, the arms exerted greater effort when using the arm-leg pulley system but the arms' swinging motion (excessive shoulder protraction and elbow extension) deviates from the normal range of motion observed during normal walking. It will be important to understand whether this excessive type of arm swinging motion would exploit, or possibly disrupt, the neural linkage that underlies the coordinated motion of the arms and legs during walking.

## Conclusions

In summary, we found that our simple method of physically linking the arms and legs during walking did not assist leg swing as expected. Instead, we found that through our arm and leg rope connection, subjects could harness the energy from the motor-driven treadmill belt to help propel the whole body during walking. The forward assistance at the whole body helped reduce the need of the legs to generate propulsive forces, leading to a significant reduction in the net metabolic power required to walk. Our data indicate that the arms played an active role in helping propel the whole body and a key design feature of our arm-leg pulley system is that the users themselves can elicit a mechanical and muscular shift that requires a unique trade-off between the active use of the arms and legs. The findings presented here provide the biomechanical and energetic basis for how we might reimagine the active use of the arms in gait rehabilitation, opening the opportunity to explore if such an arm-leg pulley system could help patients regain their walking ability.

## Supplementary Information

**Additional file 1.** Photo of arm-leg rope pulley system, split-belt treadmill, and user. This photo was taken during pilot testing where we developed the standard protocol to attach the device to each subject (as described in the methods section). Note that the reflective markers, EMG sensors, load cells, and metabolic cart are not shown here. Also note that all of our subjects were instructed to wear shorts and a tank top that would allow for proper placement of the EMG sensors and reflective markers on the body.

**Additional file 2: Figure S1.** Mean ensemble curves for each subject. Description: This figure contains the mean ensemble curves of assistive force, horizontal GRF, vertical GRF, relative joint angles (shoulder, elbow, hip, knee and ankle) for each subject.

**Additional file 3: Table S1.** Average EMG data for subjects during the braking, propulsive and swing phase of walking. This file contains tables containing descriptive and inferential statistics of the aEMG data of all muscles assessed (PD, AD, TRI, BIC, BF, RF, SOL, MG, and TA) during their respective walking phase.

**Additional file 4.** Representative subject walking with the arm-leg rope pulley system (Assisted walking). This video shows the subject as a 3-D body made by the reflective markers while also showing the right-sided rope tension (i.e. assistive force) and right force plate signals (i.e. horizontal forces generated by the right leg). The video captures one gait cycle and highlights key details from the discussion to help the viewer visualize the arm and leg rope connection and the treadmill. Note that the signal illustrating the forces generated in the anterior–posterior direction are displayed as ‘action’ forces in the software. Thus, the ‘reaction’ forces are simply opposite in direction.

## Data Availability

The datasets used and/or analysed during the current study are available from the corresponding author on reasonable request.

## Abbreviations

EMG: Electromyography
$$\dot{V}{\text{O}}_2$$: Oxygen consumption
$\dot{V}{\text{CO}}_2$: Carbon dioxide production
RER: Respiratory exchange ratio
GRF: Ground reactions force
aEMG: Average electromyography
PD: Posterior deltoid
AD: Anterior deltoid
TRI: Triceps brachii
BIC: Biceps brachii
BF: Bicep femoris
RF: Rectus femoris
SOL: Soleus
MG: Medial gastrocnemius
TA: Tibialis anterior
BW: Body weight
