# Supplementary figures and images for: Using a simple rope-pulley system that mechanically couples the arms, legs, and treadmill reduces the metabolic cost of walking
Source: J Neuroeng Rehabil. 2021 Jun 7;18:96. doi: 10.1186/s12984-021-00887-3 (PMC8186224; doi:10.1186/s12984-021-00887-3)

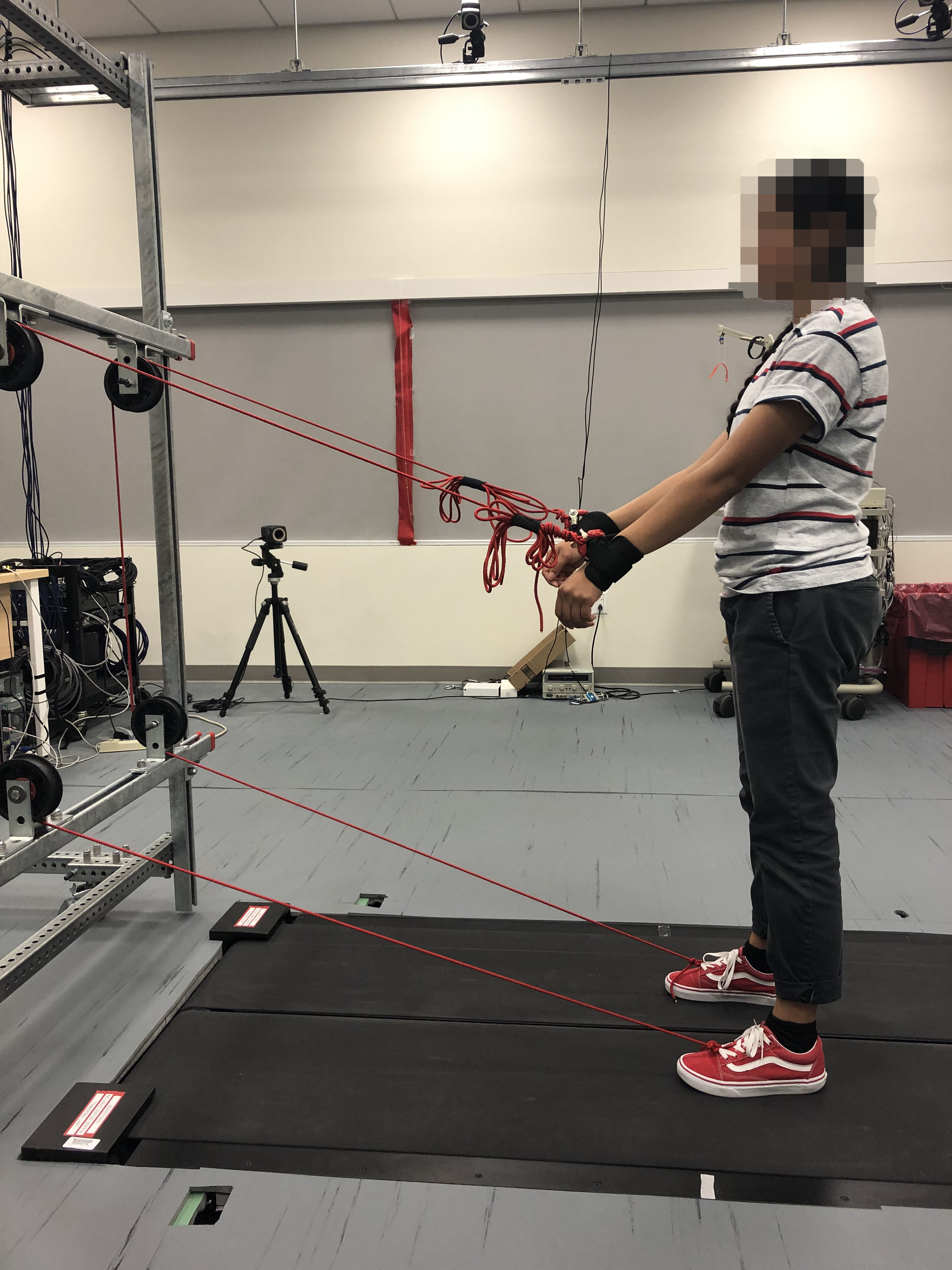

Supplement: Supplementary file 1 — Additional file 1. Photo of arm-leg rope pulley system, split-belt treadmill, and user. This photo was taken during pilot testing where we developed the standard protocol to attach the device to each subject (as described in the methods section). Note that the reflective markers, EMG sensors, load cells, and metabolic cart are not shown here. Also note that all of our subjects were instructed to wear shorts and a tank top that would allow for proper placement of the EMG sensors and reflective markers on the body. [file 12984_2021_887_MOESM1_ESM.jpg]
