# Supplementary material for: Using a simple rope-pulley system that mechanically couples the arms, legs, and treadmill reduces the metabolic cost of walking
Source: J Neuroeng Rehabil. 2021 Jun 7;18:96. doi: 10.1186/s12984-021-00887-3 (PMC8186224; doi:10.1186/s12984-021-00887-3)
Supplement: Supplementary file 2 — Additional file 2: Figure S1. Mean ensemble curves for each subject. Description: This figure contains the mean ensemble curves of assistive force, horizontal GRF, vertical GRF, relative joint angles (shoulder, elbow, hip, knee and ankle) for each subject. [file 12984_2021_887_MOESM2_ESM.pdf]

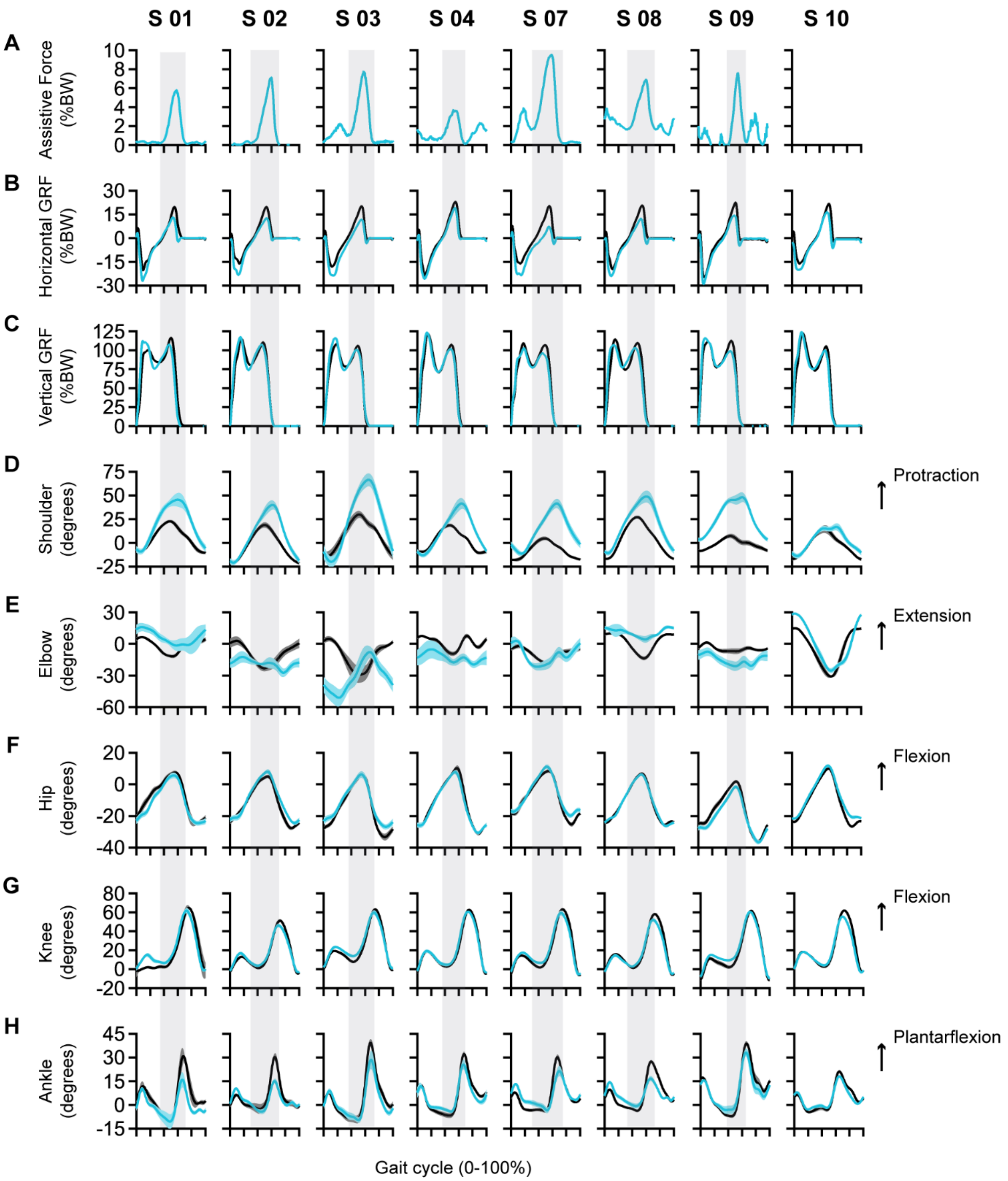

Fig. 1. Mean ensemble curves for each subject (n = 8). The blue and black lines represent the assisted and normal walking condition, respectively. (A) During assisted walking, there is a clear rise and fall in the load cell force measured at the wrist-to-rope connection (light gray shaded regions). Note that subject 10 had no load cell data due to hardware malfunction. (B) When compared to normal walking, the assistive force caused an increase in braking and decrease in propulsive ground reaction forces (GRF) for all subjects while (C) the vertical GRF remained relatively the same. (D-H)

The relative angles for the upper and lower limb were calculated and plotted as a percent of the walking gait cycle. (D) During the period of the assistive force (light gray shaded regions), the subject's arm swept a greater angle as it swung forward (increased protraction) during assisted walking as compared to normal walking. (E) While the upper arm was sweeping a greater angle as it swung forward, the subjects' elbow was undergoing either increased extension, flexion or stayed relatively fixed. (F-H) The hip and knee joint angle remained relatively the same for all subjects during both conditions while the ankle joint angle showed a decrease in max plantar flexion. Note that during the generation of peak assistive force measured at the wrist-to-rope connection, the leg was moving backward with the motor-driven treadmill belt, as indicated by hip extension.
