# Supplementary material for: Using a simple rope-pulley system that mechanically couples the arms, legs, and treadmill reduces the metabolic cost of walking
Source: J Neuroeng Rehabil. 2021 Jun 7;18:96. doi: 10.1186/s12984-021-00887-3 (PMC8186224; doi:10.1186/s12984-021-00887-3)
Supplement: Supplementary file 3 — Additional file 3: Table S1. Average EMG data for subjects during the braking, propulsive and swing phase of walking. This file contains tables containing descriptive and inferential statistics of the aEMG data of all muscles assessed (PD, AD, TRI, BIC, BF, RF, SOL, MG, and TA) during their respective walking phase. [file 12984_2021_887_MOESM3_ESM.pdf]

Table 1. Average EMG data for subjects during the braking phase of walking.

Variable	Normal mean ± SD	Assisted mean ± SD	% change	*n*	*p* value
Posterior Deltoid (mV)	0.002 ± 0.011	0.011 ± 0.006		*n* = 8	n/a
Normalized	1.000 ± 0.000	1.918 ± 1.650	91.77	*n* = 8	0.160
Anterior Deltoid (mV)	0.008 ± 0.008	0.019 ± 0.014		*n* = 8	n/a
Normalized	1.000 ± 0.000	2.996 ± 2.353	199.55*	*n* = 8	0.048
Triceps (mV)	0.005 ± 0.002	0.010 ± 0.004		*n* = 8	n/a
Normalized	1.000 ± 0.000	2.060 ± 0.479	105.99*	*n* = 8	0.000
Biceps (mV)	0.006 ± 0.003	0.019 ± 0.012		*n* = 8	n/a
Normalized	1.000 ± 0.000	3.390 ± 1.988	238.99*	*n* = 8	0.011
Bicep femoris (mV)	0.025 ± 0.0137	0.027 ± 0.018		*n* = 8	n/a
Normalized	1.000 ± 0.000	1.028 ± 0.221	2.78	*n* = 8	0.732
Rectus femoris (mV)	0.053 ± 0.039	0.050 ± 0.052		*n* = 8	n/a
Normalized	1.000 ± 0.000	0.932 ± 0.365	-6.75	*n* = 8	0.617
Soleus	0.061 ± 0.024	0.069 ± 0.030		*n* = 8	n/a
Normalized	1.000 ± 0.000	1.145 ± 0.177	14.50	*n* = 8	0.054
Medial gastrocnemius (mV)	0.048 ± 0.014	0.048 ± 0.020		*n* = 8	n/a
Normalized	1.000 ± 0.000	1.012 ± 0.275	1.19	*n* = 8	0.906
Tibialis anterior (mV)	0.031 ± 0.009	0.035 ± 0.010		*n* = 8	n/a
Normalized	1.000 ± 0.000	1.137 ± 0.115	13.72*	*n* = 8	0.012

All comparisons were made between normal and assisted walking conditions with significance at $P < 0.05$. *signifies significant differences between walking conditions. All comparisons were tested at an alpha level of 0.05 using a two-sided, paired samples t-test or a Related Samples Wilcoxon Signed Rank indicated by †. n/a = Not Applicable.

Table 2. Average EMG data for subjects during the propulsive phase of walking.

Variable	Normal mean ± SD	Assisted mean ± SD	% change	*n*	*p* value
Posterior Deltoid (mV)	0.036 ± 0.019	0.036 ± 0.025		*n* = 8	n/a
Normalized	1.000 ± 0.000	1.107 ± 0.604	10.71	*n* = 8	0.631
Anterior Deltoid (mV)	0.013 ± 0.007	0.017 ± 0.012		*n* = 8	n/a
Normalized	1.000 ± 0.000	1.488 ± 0.694	48.85	*n* = 8	0.087
Triceps (mV)	0.010 ± 0.005	0.022 ± 0.011		*n* = 8	n/a
Normalized	1.000 ± 0.000	2.317 ± 1.045	131.71*	*n* = 8	0.009
Biceps (mV)	0.006 ± 0.003	0.022 ± 0.018		*n* = 8	n/a
Normalized	1.000 ± 0.000	4.092 ± 4.294	309.18*	*n* = 8	0.017†
Soleus	0.068 ± 0.025	0.043 ± 0.018		*n* = 8	n/a
Normalized	1.000 ± 0.000	0.622 ± 0.121	-37.82*	*n* = 8	0.000
Medial gastrocnemius (mV)	0.054 ± 0.022	0.028 ± 0.020		*n* = 8	n/a
Normalized	1.000 ± 0.000	0.480 ± 0.132	-51.97*	*n* = 8	0.000

All comparisons were made between normal and walking conditions with significance at $P < 0.05$. *signifies significant differences between walking conditions. All comparisons were tested at an alpha level of 0.05 using a two-sided, paired samples t-test or a Related Samples Wilcoxon Signed Rank indicated by †. n/a = Not Applicable.

Table 3. Average EMG data for subjects during the swing phase of walking.

Variable	Normal mean ± SD	Assisted mean ± SD	% change	n	*p* value
Posterior Deltoid (mV)	0.021 ± 0.015	0.018 ± 0.009		*n* = 8	n/a
Normalized	1.000 ± 0.000	1.580 ± 1.580	58.03	*n* = 8	0.445†
Anterior Deltoid (mV)	0.012 ± 0.010	0.020 ± 0.011		*n* = 8	n/a
Normalized	1.000 ± 0.000	2.251 ± 1.240	125.15*	*n* = 8	0.013
Triceps (mV)	0.007 ± 0.004	0.013 ± 0.007		*n* = 8	n/a
Normalized	1.000 ± 0.000	2.470 ± 1.657	146.96*	*n* = 8	0.020
Biceps (mV)	0.007 ± 0.003	0.033 ± 0.020		*n* = 8	n/a
Normalized	1.000 ± 0.000	5.203 ± 2.110	420.27*	*n* = 8	0.001
Bicep femoris (mV)	0.023 ± 0.009	0.019 ± 0.006		*n* = 8	N/A
Normalized	1.000 ± 0.000	0.891 ± 0.258	-10.87	*n* = 8	0.081†
Rectus femoris (mV)	0.021 ± 0.024	0.027 ± 0.027		*n* = 8	n/a
Normalized	1.000 ± 0.000	1.502 ± 1.366	50.16	*n* = 8	0.288†
Tibialis anterior (mV)	0.064 ± 0.024	0.067 ± 0.023		*n* = 8	n/a
Normalized	1.000 ± 0.000	1.058 ± 0.142	5.79	*n* = 8	0.145

All comparisons were made between normal and assisted walking conditions with significance at $P < 0.05$. *signifies significant differences between walking conditions. All comparisons were tested at an alpha level of 0.05 using a one-sided, paired samples t-test or a Related Samples Wilcoxon Signed Rank test indicated by †. n/a = Not Applicable.
